# Structure, Physicochemical Property, and Functional Activity of Dietary Fiber Obtained from Pear Fruit Pomace (*Pyrus ussuriensis* Maxim) via Different Extraction Methods

**DOI:** 10.3390/foods11142161

**Published:** 2022-07-21

**Authors:** Fei Peng, Xin Ren, Bin Du, Linan Chen, Zuoqing Yu, Yuedong Yang

**Affiliations:** 1 Hebei Key Laboratory of Active Components and Functions in Natural Products, Hebei Normal University of Science and Technology, Qinhuangdao 066004, China; feipeng1986@hotmail.com (F.P.); rx120593131@163.com (X.R.); bindufood@aliyun.com (B.D.); chen15534445342@163.com (L.C.); jesus_yu@163.com (Z.Y.); 2 Engineering Research Center of Chestnut Industry Technology, Ministry of Education, Qinhuangdao 066004, China

**Keywords:** *Pyrus ussuriensis* Maxim, dietary fiber, structure composition, functional activity

## Abstract

In this study, soluble dietary fiber (SDF) and insoluble dietary fiber (IDF) were extracted from Pyrus ussuriensis Maxim pomace via three methods including enzymic extraction (EE), microwave-assisted enzymatic extraction (MEE), and three-phase partitioning (TPP). The effects of different extraction methods on the structure, physicochemical property, and functional activity of the extracted dietary fiber were evaluated. The results showed that different extraction methods had significant effects on the extraction yield, molecular weight distribution, thermal stability, antioxidant activity, and hypoglycemic activity in vitro, but resulted in no difference in the structure and composition of functional groups. It is noteworthy that SDF extracted by TPP has a more complex and porous structure, lower molecular weight, and higher thermal stability, as well as better physicochemical properties and in vitro hypoglycemic activity. IDF extracted by MEE showed the greatest water and oil holding capacity; the highest adsorption capacity for glucose, cholesterol, and nitrite ion; as well as the strongest inhibitory activity on α-amylase. These results suggest that PUP may be a source of cheap natural dietary fiber.

## 1. Introduction

Dietary fiber (DF) is a complex carbohydrate with polymerization degrees >10 in plants [1]. According to its water solubility, DF can be divided into soluble dietary fiber (SDF) and insoluble dietary fiber (IDF) [2]. SDF includes pectin, β-glucan, and fructan, which are abundant in fruits [3], vegetables [4], and oats [5]. IDF comprises cellulosic lignin [6] and some hemicellulose, and is abundant in grains [7]. The complex spatial structure and abundant active groups endow DF with a higher water holding capacity and adsorption capacity for small molecules. Previous research has demonstrated that DF can regulate blood glucose [8,9] and the concentration of blood lipids [10], improve drug tolerance [11], reduce total cholesterol and lower the density of lipoprotein cholesterol [12], and maintain intestinal health and reduce the risk of cancer and other chronic diseases [13,14]. Supplementation of an appropriate amount of DF to food can improve the taste of food and indirectly improve human health.

At present, many extraction methods of DF are applied in the food production industry, including chemical methods, biological methods, and physical methods. The chemical methods employ acids and bases to dissolve the impurities to extract DF, which has the advantages of simple operation and low preparation cost. However, it easily results in chemical reagent residue, and leads to the loss of hemicellulose and cellulose in the raw materials. The biological methods make use of specific enzymes or microorganisms for the enzymolysis or fermentation of DF into small molecules. As a new technology, physical extraction has been widely used in the extraction of nutritional/functional compounds, which can effectively enhance the proportion of SDF in total DF. Microwave extraction utilizes the water molecules in the cell to absorb the heat generated by microwaves, leading to an increase in intracellular temperature and vaporization of liquid water to break the cell membrane/wall and form fine pores, which will allow the dissolution and release of intracellular substances [15]. Three-phase partitioning (TPP) is a new non-chromatographic biological separation method, whose principle is to separate and extract small organic compounds from protein lipids and oils by salting out isoelectron precipitation and co-solvent precipitation in tert-butanol and ammonium sulfate solutions [16]. This method has the characteristics of high extraction efficiency and low energy consumption and can retain the physiological activity of raw materials.

*Pyrus ussuriensis* Maxim originated from China with a long history of planting and has entered a period of scale development. This fruit is coarse in taste but rich in pectin and stone cells. With the rapid development of the fruit processing industry such as fruit juice and jam, large quantities of fruit pomace will be generated in the production process. After pressing, pomace accounts for nearly 40% of the whole fruit mass of pear, and DF accounts for about 75% of the pomace on a dry basis. Fruit pomace comprises a large amount of stone cells [17], mainly including lignin and cellulose, which can be high-quality raw materials for the production of DF. At present, studies of pears are mainly focused on small-molecule components such as polyphenols [18], while there have been few studies on the extraction of large-molecule components such as DF. This study used enzymic extraction (EE), microwave-assisted enzymatic extraction (MEE), and three-phase partitioning (TPP) to extract SDF and IDF from PUP, and analyzed the influence of different methods on the structure, physicochemical property, and functional activity of the extracted SDF and IDF. The findings may provide a theoretical basis for further research on DF from PUP and related product development.

## 2. Materials and Methods

### 2.1. Reagents

*Pyrus ussuriensis* Maxim pear fruit were collected from Qinglong of Hebei Province. Heat-stable α-amylase, amyloglucosidase, and neutral protease were purchased from Guangzhou Qiyun Biotechnology Co., Ltd. (Guangzhou, China). 1,1-diphenyl-2-picrylhydrazine (DPPH), 3,5-dinitrosalicylic acid (DNS), the cholesterol content (phthalaldehyde method) determination kit, the ABTS free radical scavenging ability test kit, and the total antioxidant capacity test kit (FRAP method) were purchased from Beijing Solaibao Technology Co., Ltd. (Beijing, China).

### 2.2. Preparation of Pomace

Mature pear fruit were washed and cut into small pieces of uniform size, and smashed in a GM-200 knife grinding mill. Then, the fruit pomace and juice were separated with gauze and vacuum freeze-dried. After grinding, the powder sample was obtained through a sieve of 60 mesh to obtain *P. ussuriensis* pomace powder (PUPP), which was frozen and stored in the refrigerator for later use.

### 2.3. Preparation of Samples

**Figure 1:**
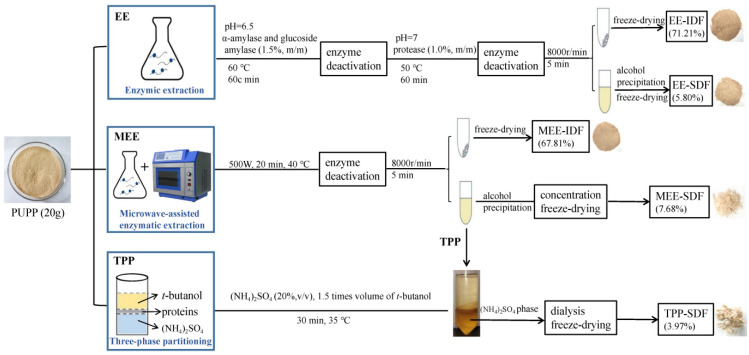
Schematic procedures for the extraction of dietary fiber from PUP.

The SDF and IDF in PUP were extracted by EE, MEE, and TPP. The specific extraction processes are presented in Figure 1.

For EE, the PUPP was mixed with water at a ratio of 1:20, and the pH was adjusted to 6.5. Then, 1.5% high-temperature α-amylase and glucoside amylase were added and enzymolyzed at 60 °C for 1 h. Subsequently, the enzyme was denatured by a boiling water bath. The solution pH was adjusted to 7.0, followed by the addition of 1% protease, Then, the solution was enzymolyzed at 50 °C for 1 h, and the enzyme was removed by a boiling water bath. After cooling, the mixture was centrifuged (8000 r/min, 5 min) to collect the precipitate, which was denoted as EE-IDF. The supernatant was precipitated by four volumes of 95% ethanol for 12 h. Subsequently, the precipitate was collected by centrifugation and freeze-drying, which was denoted as EE-SDF.

For MEE, 20 g of PUPP was mixed with 400 mL of deionized water. The pH was adjusted to 6.5. Then, 1.5% high-temperature α-amylase and glucoside amylase were added and stirred for 60 min at 60 °C. The mixture was placed in a microwave chemical reaction synthesizer (microwave power, 500 W) for extraction for 20 min at 30 °C. Subsequently, the pH was adjusted to 7. Protease (1%, *m*/*v*) was added and stirred for 60 min at 50 °C and then the mixture was treated with the same microwave conditions again. The remaining steps were the same as the EE method to obtain the SDF (MEE-SDF) and IDF (MEE-IDF).

For TPP, the supernatant obtained by MEE was concentrated to half of its original volume and mixed with 20% (*w*/*w*) (NH4)_2_SO_4_ under continuous stirring. Then, a 1.5-fold volume of tert-butanol was added after full mixing. The mixture was continuously agitated by an electronic stirrer for 30 min at 35 °C. After centrifugation (8000 r/min, 5 min), the lower (NH4)_2_SO_4_ phase was collected and dialyzed in a 3500 Da dialysis bag for 48 h, and then concentrated and lyophilized to obtain water-soluble dietary fiber designated as TPP-SDF.

### 2.4. Routine Constituents

The routine constituents of samples were determined according to the national standards of ash content in food (GB5009.4-2016), the determination of moisture in food (GB5009.3-2016), and the determination of protein in food (GB5009.5-2016). The total sugar content was analyzed by AOAC 968.28 [19]. The dietary fiber content was determined by AOAC 991.43 (AOAC method, 1996) [20].

### 2.5. Physicochemical Property Analysis

#### 2.5.1. Water Holding Capacity (WHC), Water Binding Capacity (WBC), and Oil Holding Capacity (OHC)

According to the method of Ma et al. [21], 0.5 g of the sample (M_0_) was placed in a test tube, followed by the addition of 30 mL of distilled water/peanut oil. The mixture was well shaken and let to stand at room temperature for 24 h, followed by centrifugation at 4000 r/min for 20 min. Then, the mass was determined after removal of the supernatant (M_1_). The WBC test was carried out in the next step. After the sample was centrifuged, the weight was recorded as M_2_. Then, the sample was dried for 2 h at 120 °C, and the weight was recorded as M_3_. The specific calculation formula is as follows:

WHC (g/g) = [(M_1_ − M_0_)/M_0_], (1)

OHC (g/g) = [(M_1_ − M_0_)/M_0_], (2)

WBC(g/g) = (M_2_ − M_3_)/M_2_, (3)

#### 2.5.2. Water Swelling Capacity (WSC)

According to the method of Sowbhagya et al. [22], 0.5 g (M_0_) of the sample was accurately weighed and placed in a stop-graduated tube. After recording the volume (V_1_), 10 mL of distilled water was added. The mixture was well shaken and let to stand at room temperature for 24 h, and the volume of the sample was recorded after settling (V_2_).

WSC (mL/g) = (V_2_ − V_1_)/M_0_, (4)

### 2.6. Monosaccharide Composition

The monosaccharide composition of SDF was analyzed using ion chromatography analysis (ICS5000+, Thermo Fisher Scientific, Waltham, MA, USA) with the Dionex CarboPac PA10 (250 × 4.0 mm, 10 μm) liquid chromatographic column at 30 °C. The gradient eluent used was H_2_O (mobile phase A) and 100 mM NaOH (mobile phase B) with a flow rate of 0.4 mL/min. The following elution program was applied: 0–30 min, 97.5–80% A; 30–30.1 min, 80–60% A; 30.1–45 min, 60% A; 45–45.1 min, 60–97.5% A. Fucose (Fuc), arabinose (Ara), galactose (Gal), glucose (Glu), xylose (Xyl), mannose (Man), fructose (Fru), ribose (Rib), galacturonic acid (Gal-UA), Glucuronic acid (Glc-UA), and Mannuronic acid (Man-UA) were used as monosaccharide standards.

### 2.7. Molecular Weight Distribution

High-performance liquid chromatography (HPLC 1100, Agilent Technologies, Palo Alto, CA, USA) with a G1362A differential refractive index detector (RID) and Agilent PL aquagel-oh mixed-h (300 × 7.5 mm, 8 μm) gel column was used to determine the molecular weight distribution (Mw). The eluent used was H_2_O at a flow rate of 0.8 mL/min. The mass concentration of the standard and sample was 2 mg/mL, and the injection volume was 20 μL.

### 2.8. Structural Analysis

Scanning electron microscopy was performed with a Hitachi scanning electron microscope S-3400 N (Tokyo, Japan). The infrared absorption spectra were collected on a Fourier transform infrared (FT-IR) spectroscope (Bruker, Germany). Thermogravimetric analysis (TGA) was carried out with a STA 409 PC (Netzsch, Bavaria, Germany).

### 2.9. Functional Characteristics

#### 2.9.1. Antioxidant Capacity

Three methods were used to test the in vitro antioxidant capacity of the samples, including the DPPH free radical scavenging capacity, ABTS free radical scavenging capacity, and ferric reducing antioxidant power (FRAP) assay as described by Bender et al. [23]. BHT was used as the positive control in the DPPH assay. The ABTS free radical scavenging capacity was represented as mmol Trolox/g DM and FRAP was represented as FeSO_4_/mM, which were derived from the standard curve of Trolox (0.05–1.2 mM) and FeSO_4_ (0.05–1.5 mmol/L), respectively.

#### 2.9.2. Cholesterol Adsorption Capacity (CAC)

According to the method of Jia et al. [24], 0.5 g (M_0_) of the sample was mixed with 25 mL of diluted yolk (water: yolk = 9:1) and the pH was adjusted to 2.0 or 7.0. The mixture was oscillated in a 37 °C water bath for 30, 60, 90, and 120 min, and then centrifuged at 10,000 r/min for 10 min. The cholesterol content in the supernatant was determined by the cholesterol assay kit.

CAC (mg/g) = (M_1_ − M_2_)/M_0_, (5)

where M_1_ is the amount of cholesterol in the original solution (mg), and M_2_ indicates the amount of cholesterol in the solution after adsorption (mg).

#### 2.9.3. Nitrite Adsorption Capacity (NAC)

According to the method of Hua et al. [25], 0.2 g (M_0_) of the sample was mixed with 100 mL of NaNO_2_ (20 μg/mL) and the pH was adjusted to 2.0 or 7.0. The mixture was oscillated in a 37 °C water bath for 20 min. Then, 1 mL of the supernatant was taken and mixed with 2 mL of *p*-aminobenzenesulfonic acid (4 g/L) in a 10 mL volumetric flask, well shaken, and allowed to stand for 3–5 min. Then, 1 mL of naphthalenediamine hydrochloride (2 g/L) was added, followed by the addition of water to scale and standing for 15 min. The absorbance was measured at 538 nm.

NAC (μg/g) = (C_1_ − C_2_)V/M_0_, (6)

where C_1_ and C_2_ are the NO^2−^ concentration in the solution before and after adsorption (μg/mL), respectively; V is the volume of the tested sample.

#### 2.9.4. Glucose Adsorption Capacity (GAC)

According to the method of Peerajit et al. [26], 20 mL of glucose solution at different concentrations (50–200 mmol/L) was mixed with 0.5 g (M_0_) of PUPP and oscillated at 37 °C for 4 h, followed by centrifugation at 10,000 r/min for 10 min. The glucose content in the supernatant was detected at 490 nm. GAC was calculated with the following equation:

GAC (mmol/g) = (C_1_ − C_2_)/M_0_, (7)

where C_1_ and C_2_ are the amount of glucose in the original solution and after adsorption (mmol), respectively.

#### 2.9.5. Inhibitory Effect of SDF on α-Amylase (α-AAIR)

As described previously by Benítez et al. [27], 40 mL of soluble starch solution (1%, *w*/*v*) was mixed with 0.5 g of the sample and 4 mg of α-amylase and incubated for 30 min at 37 °C. Then, 2.0 mL of 0.1 N NaOH was added to stop each reaction. The mixture was centrifuged at 3500 r/min for 15 min, and the glucose content in the supernatant was determined by the DNS method with the sample without SDF as the control. A-AAIR was calculated with the following equation:

α-AAIR (%) = (C_2_ − C_1_)/C_2_ × 100% (8)

where C_1_ and C_2_ are the concentration of glucose in the experimental group (α-amylase + sample + soluble starch) and the control group (α-amylase + soluble starch) (mmol), respectively.

### 2.10. Statistical Analysis

The SPSS 22.0 version (SPSS Inc., Chicago, IL, USA) was used to analyze the Pearson correlation coefficients. The significant differences of data were determined for comparative analysis using Duncan’s multiple range test. Statistically, *p* < 0.05 indicates significant differences.

## 3. Results and Discussion

### 3.1. Proximate Composition

The proximate composition of SDF and IDF samples from PUP and the relative contents of each component are presented in Table 1. The relative content of total DF in PUP was 79.9 ± 1.02 g/100 g, which was higher than those in pear residue (56.9 g/100 g) [28] and rice bran (27.04 g/100 g) [29], indicating that PUP is a good source of DF. Compared with PUP, the three SDFs showed reductions in impurities such as crude protein and ash, particularly TPP-SDF, whose crude protein content was reduced by 61.5%, indicating that TPP can effectively remove impurities such as protein. In addition, the ash content increased while the protein content decreased in IDFs. MEE-SDF and TPP-SDF exhibited significant increases in total sugar content compared with PUP, which may contribute to better biological activities. Microwaves can degrade long-chain polysaccharides into short-chain polysaccharides to a certain extent and increase the total sugar content. These results are consistent with the findings in previous studies [30].

### 3.2. Physicochemical Properties

DF is a mixture of complex carbohydrates with a polysaccharide structure. The physical properties of DF, such as good water holding, viscosity, binding, or adsorption, contribute to its desirable physiological functions. The hydraulic and expansive force is an important index to measure the quality of DF, and its higher value generally indicates better adsorption performance [31], because DF has a large number of hydrophilic groups, which can bind with water molecules and expand to produce a volume effect, improve satiety, decrease food intake, and reduce obesity [32]. As shown in Table 1, the WHC, WSC, and OHC of MEE-IDF were significantly increased compared with those of EE-IDF. WHC, WBC, and WSC values of TPP-SDF were highest, especially with a WHC as high as 5.01 g/g, which is significantly higher than those of SDFs from oat husk (2.13 g/g), bagasse (3.68 g/g), and rice husk (2.58 g/g) [33]. These results demonstrated that TPP can confer the extracted SDF with more hydrophilic groups and an improvement of water absorption. On the other hand, OHC is an important indicator of DF quality, which can help maintain the stability of high-fat foods. Table 1 shows that MEE-IDF had the highest OHC, which was significantly higher than those of IDF from papaya skin (1.4 g/g) [34], rice husk (1.85 g/g), malted bagasse (2.46 g/g), and oat husk (1.37 g/g) [33].

### 3.3. Monosaccharide Composition of SDFs

As shown in Table 2, the SDF of PUP was mainly composed of galactose and galacturonic acid. When treated with microwaves and enzymes, the glucose ratio decreased from 33.11% to 21.11% and 16.99%, respectively, while the galacturonic acid ratio showed a significant increase. It is possible that the enzyme degrades the macromolecules such as glucan starch in PUP to produce more small molecules, thus improving the yield of SDF. Compared with the other two methods, TPP could generate a higher proportion of arabinose and mannose, and a small amount of mannuronic acid, indicating that TPP is a soft extraction method, which is conducive to the better retention of nutrients and may confer the extracted SDF with better functional activity [35].

### 3.4. Structural Characterization of SDFs

**Figure 2:**
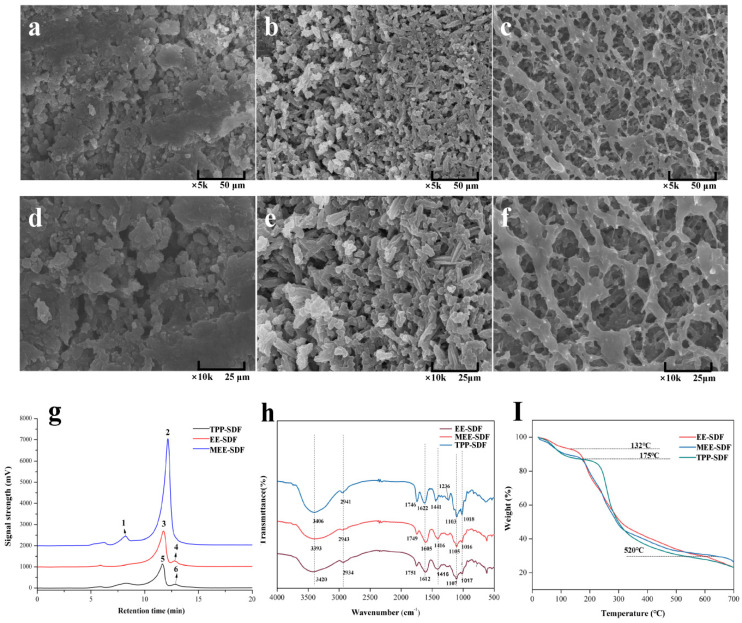
Structural characteristics of SDFs prepared with different extraction methods. SEM of EE-SDF × 5 k (**a**); MEE-SDF × 5 k (**b**); TPP-SDF × 5 k (**c**); EE-SDF × 10 k (**d**); MEE-SDF ×10 k (**e**); TPP-SDF ×10 k (**f**); molecular weight distribution (**g**); FT-IR spectra of SDFs (**h**); TGA curves of SDFs (**I**).

Figure 2a–e show the SEM images of SDF obtained with different extraction methods. It can be seen that EE-SDF showed a shape of irregular blocks (Figure 2a,d), while MEE-SDF presented a uniform spindle shape (Figure 2b,e), which may be due to the reduction in SDF particle size caused by the co-decomposition effect of enzymes and microwaves in the extraction process [36]. In addition, TPP-SDF obviously had a large pore and network structure (Figure 2c,f), which can greatly increase the surface area, promote water absorption and expansion, and improve the ability of the SDF to absorb glucose, cholesterol, and harmful substances in the gastrointestinal tract.

The molecular weights (Mw, Mn, and Mw/Mn) of the three SDFs were determined by the SEC-MALLS analysis method. The results are presented in Table 3 and Figure 2g. Different extraction methods resulted in significant differences in the molecular weights of SDFs. It has been reported that different extraction methods tend to result in different molecular weights, which can further affect the functional activity of SDF [37]. EE-SDF, MEE-SDF, and TPP-SDF showed two main peaks, in which the Mw value of the highest peak was 168.57, 9.28, and 2.66 kDa, and the peak area was 87.91%, 92.90%, and 86.10%, respectively (Table 3), indicating that microwave treatment can facilitate the release of SDF with low molecular weights, which is similar to the effect of microwave treatment reported by Yang et al. [38]. In addition, the TPP-SDF had lower Mw values than MEE- and EE-SDF, mainly because TPP extraction tends to result in low molecular weights of SDF. Previous studies have shown that DF with low molecular weights can more significantly reduce the absorption of cholesterol and promote its excretion in feces [39]. A lower polydispersity coefficient usually represents a more concentrated and uniform distribution of the molecular weight. Peak 5 shows that TPP-SDF has a relatively low polydispersity coefficient (Mw/Mn = 2.66), indicating that it has a relatively concentrated and uniform distribution of molecular weight.

The infrared spectra (Figure 2h) show that the three SDFs had no significant difference in the number and position of peaks, indicating that they have similar main components and overall chemical structure. The strong peak near 3400 cm^−1^ can be attributed to the stretching vibration of the O-H bond, and the rounded peak indicates that there are more hydroxyl groups and bound water molecules in SDF. The peak near 2940 cm^−1^ corresponds to the C-H stretching vibration of methyl and methylene. The absorption peaks at 1749 cm^−1^ and 1620 cm^−1^ are characteristic of carboxyl or aldehyde groups, indicating that SDF contains a certain amount of aldehyde acids. The peak at 1416 cm^−1^ indicates the bending vibration of the C-H bond; there is a narrow and strong peak at 1016 cm^−1^, which can be ascribed to the C-O-C bond stretching vibration of glucopyranoside. These peaks are typical absorption peaks for polysaccharides. TPP-SDF had the largest O-H peak area, indicating that it has more intramolecular hydrogen bonds and, therefore, higher hydrophilicity. Yan [40] also reached a similar conclusion by using TPP to extract polysaccharides from bitter melon. Therefore, TPP may be an effective method to extract DF with higher activity.

TG analyses were used to evaluate the changes in the thermal stability and structural properties of DF. As shown in Figure 2I, there were significant changes in the mass of SDF in three temperature ranges (30–200 °C, 200–500 °C, and 500–700 °C), exhibiting great differences in the degradation rate of SDFs. These results indicate that different temperatures will lead to different types of pyrolytic products, and the maximum weight loss occurs in the temperature range of 200–300 °C. The first degradation temperature range can be attributed to the weight loss of the bound water in the sample. In addition, MEE-SDF and TPP-SDF showed a greater mass loss than EE-SDF, indicating that the SDF obtained by MEE and TPP had more bound water than EE-SDF. Moreover, TPP-SDF had a higher degradation temperature than other two SDFs, indicating that TPP-SDF had a stronger bond between molecules and water. This is due to the increased number of short chains in TPP-SDF and stronger hydrogen bonds at binding sites, which require higher energy to destroy its structure [41]. In the second temperature range, a large amount of weight loss occurred in SDF, which is associated with the degradation of pectin, hemicellulose, and other sugars in SDF at this stage. When the pyrolysis temperature increased to the third temperature range, the mass loss became slower, which may be ascribed to the thermal decomposition of carbon.

### 3.5. Functional Characteristics of SDFs

#### 3.5.1. Antioxidant Activities

**Figure 3:**
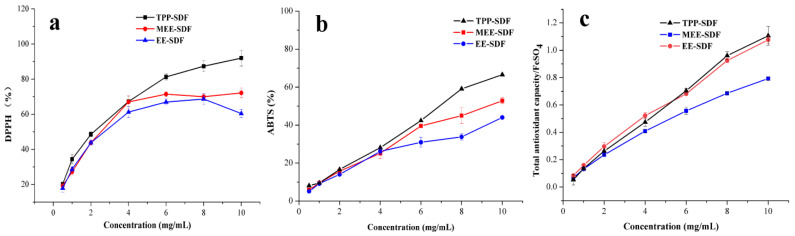
Antioxidant activity of SDFs obtained with different extraction methods. DPPH (**a**), ABTS free radical scavenging ability (**b**), and FRAP (**c**).

As shown in Figure 3, with increasing SDF concentration, the DPPH and ABTS free radical scavenging capacity and FRAP value of the sample also increased gradually and reached the maximum value at 10 mg/mL. DPPH scavenging results revealed that the scavenging rate of the three SDFs for DPPH reached about 50% at 4 mg/mL, and the increase in scavenging rate tended to be gentle at 8 mg/mL. Analysis of the three kinds of antioxidant activities demonstrated that the antioxidant capacity of SDF is concentration-dependent and follows the order of MEE-SDF < EE-SDF < TPP-SDF. This may be due to the fact that TPP-SDF possessed loose and porous structures, and reduced molecular weight and monosaccharide composition, which facilitated the donation of electrons or hydrogen atoms of polysaccharide hydroxyl groups, forming stable free radicals and terminating free radical reactions. Yan et al. [42] suggested that the composition of monosaccharides, polymerization degree, structure, molecular weight, and conformation of polysaccharides all might play important roles in the antioxidant activity. 

#### 3.5.2. Cholesterol Adsorption Capacity (CAC)

**Figure 4:**
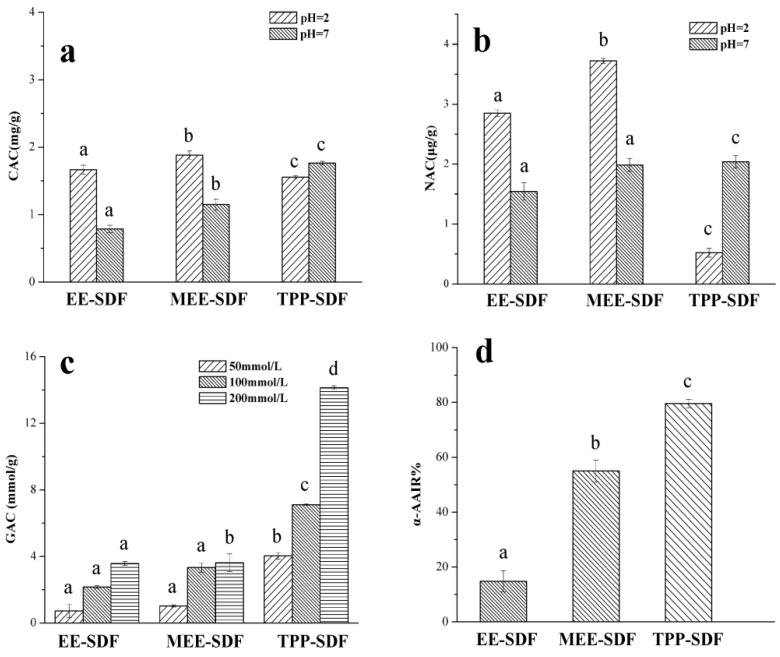
Hypoglycemic activity of SDFs obtained with different extraction methods. CAC (**a**), NAC (**b**), GAC (**c**), and α-AAIR (**d**). Different letters are statistically different (*p* < 0.05).

It is generally considered that the binding ability of dietary fiber to oil and cholesterol is an important indicator to assess its ability to absorb lipophilic components. Figure 4a shows that the cholesterol adsorption capacity of the three SDFs was 1.882, 1.666, and 1.554 mg/g at pH = 2, and 0.788, 1.148, and 1.764 mg/g at pH = 7, respectively. In particular, at pH = 2, MEE-SDF had a good adsorption effect for cholesterol, while TPP-SDF had a stronger cholesterol adsorption effect at pH = 7. As with the OHC, CAC may be associated with the properties such as porosity, available surface area, and the regiochemistry. Under acidic conditions, a large number of hydrogen ions were found in the system repulsion with a partial positive charge of dietary fiber molecules and cholesterol, resulting in a decrease in binding force. With increasing pH, the carboxyl groups in dietary fiber molecules were dissociated and converted into carboxyl anions (RCOO^−^), which have a strong binding ability with cholesterol molecules, thereby improving the adsorption capacity of dietary fiber for cholesterol [43].

#### 3.5.3. Nitrite Adsorption Capacity (NAC)

As shown in Figure 4b, the scavenging capacity of MEE-SDF on NO^2−^ at pH 2.0 was much higher than that at pH 7.0. On the contrary, the nitrite adsorption capacity of TPP-SDF was highest at pH 7.0, suggesting that the scavenging effect of MEE-SDF on NO^2-^ was mainly in the stomach, while TPP-SDF was mainly in the intestine.

#### 3.5.4. Glucose Adsorption Capacity (GAC)

The glucose absorption capacity of DF is beneficial to reduce the content of available glucose in the small intestine, thus reducing the postprandial serum glucose levels [44]. Figure 4c shows that SDFs can effectively adsorb glucose at different glucose concentrations and is positively correlated with the concentration. The glucose adsorption capacity (GAC) of TPP-SDF was significantly higher than that of other two SDFs, particularly at the concentration of 200 mmol/L, at which the GAC was as high as 14.133 mmol/g. This may also be due to an increase in the number of voids or voids on the surface of TPP-SDF (Figure 2) and its excellent hydration ability, which increases the viscosity of intestinal contents and delays the release of glucose in starch, thereby reducing blood glucose levels. It has been reported that increasing the porosity and specific surface area of dietary fiber can increase the number of glucose molecules trapped in the fiber network and thus increase its GAC [21].

#### 3.5.5. Inhibitory Effect of SDF on α-Amylase (α-AAIR)

Amylase inhibition is an important index to evaluate the ability of dietary fiber to reduce the rate of glucose production in vitro. Figure 4d shows that all three types of SDFs could reduce α-amylase activity, although to varying degrees. The α-amylase inhibition activity of TPP-SDF is best, possibly due to the porous structure of TPP-SDF, which is conducive to adsorption on starch and hinders its binding to the active site of α-amylase [45].

### 3.6. Structural Characteristics of IDFs

**Figure 5:**
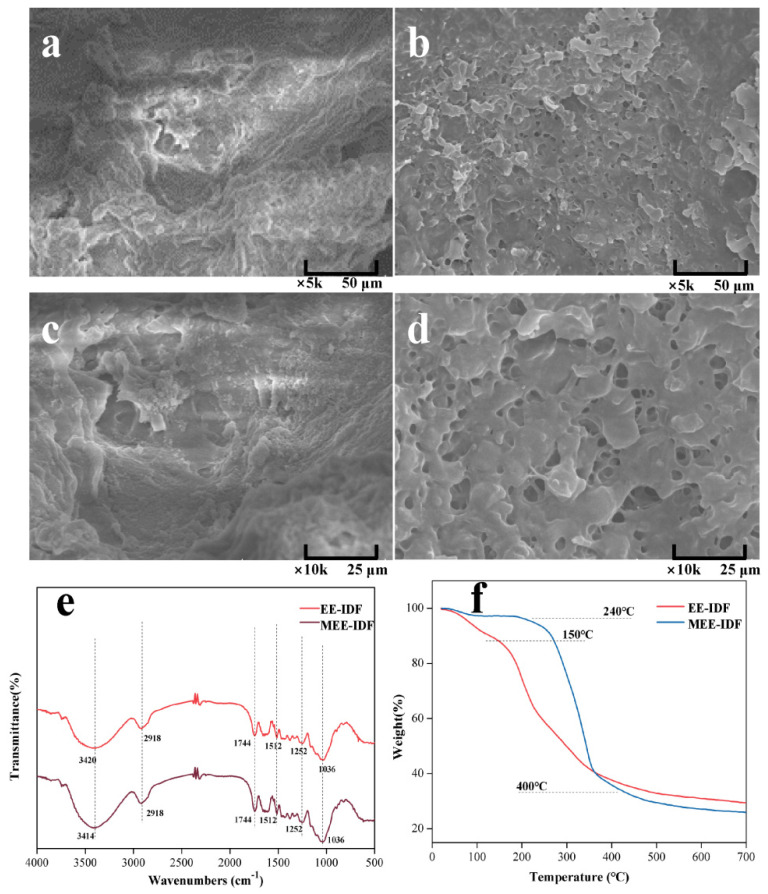
Structural characteristics of IDFs prepared with different extraction methods. SEM of EE-IDF × 5 k (**a**); MEE-IDF × 5 k (**b**); EE-IDF × 10 k (**c**); MEE-IDF × 10 k (**d**); FT-IR spectra of IDFs (**e**); TGA curves of IDFs (**f**).

As shown in Figure 5a–d, EE-IDF has complex multilayer folds and a certain fiber structure. There are many holes on the surface of MEE-IDF, which may be caused by the cavitation effect of microwaves in the extraction process [46], indicating that enzymes and microwaves could work synergistically to dissolve part of the substances in the cell wall of pear residue and increase the specific surface area of IDF, which might affect its physical and chemical properties and adsorption capacity.

The infrared spectra (Figure 5e) show that the absorption peak of IDFs near 3420 cm^−1^ can be assigned to the stretching vibration peak of -OH, which is the characteristic band of natural cellulose. The absorption peak at 2918 cm^−1^ represents the stretching vibration peak of aliphatic saturated C-H in cellulose and hemicellulose. The peaks at 1744 cm^−1^ and 1659 cm^−1^ can be attributed to the unique acetyl group or C=O of hemicellulose and the methylated or free carboxyl group vibration of pectin. The bending vibration of aliphatic or aromatic C-H groups of lignin at 1512 cm^−1^ and 1433 cm^−1^ was observed. The weak peaks at 1379 cm^−1^ and 1252 cm^−1^ are characteristic peaks of O-H or C-O groups of cellulose and hemicellulose, respectively. The absorption peak at 1036 cm^−1^ is the C-O-O stretching vibration peak of cellulose and hemicellulose. The above results showed that the infrared spectra of IDF obtained by EE and MEE had no significant difference, which did not affect the structure and composition of the functional group of IDF.

As shown in Figure 5f, the weight loss of IDFs mainly underwent three stages. The first stage occurred at about 50–200 °C with a weight loss of about 3.49%, which can be attributed to the evaporation of water molecules adsorbed on the fiber surface and the presence of some compounds with low decomposition temperature. The second stage occurred between 200 and 350 °C with a weight loss of approximately 56.32%, which could be ascribed to the pyrolysis and decomposition of most soluble substances and hemicellulose polysaccharides and the transfer of some volatile components. The third stage was at 350–650 °C with a weight loss of about 14.84%, which can be attributed to the decomposition of cellulose and lignin. Compared with EE-IDF, MEE-IDF showed a significantly lower decomposition rate at 200–400 °C, as well as significantly higher decomposition temperature, indicating that the MEE-IDF is thermally more stable, which is conducive to production and processing.

### 3.7. Functional Activity of IDFs

**Figure 6:**
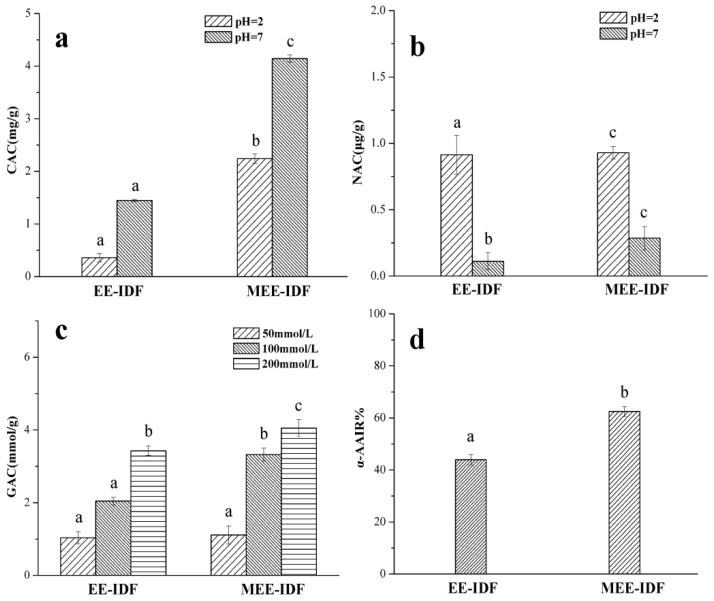
Hypoglycemic activity of IDFs obtained with different extraction methods. CAC (**a**), NAC (**b**), GAC (**c**), and α-AAIR (**d**). Different letters are statistically different (*p* < 0.05).

As shown in Figure 6a, the cholesterol adsorption capacity of IDFs obtained by EE and MEE was 1.448 and 4.142 mg/g at pH = 7, and 0.258 and 2.242 mg/g at pH = 2, respectively, indicating that EE-IDF and MEE-IDF have a higher cholesterol adsorption capacity in the intestinal tract. As presented in Figure 6b, the nitrite adsorption capacity of EE-IDF and MEE-IDF was 0.913 and 0.930 μg/g at pH = 2, and 0.112 and 0.287 μg/g at pH = 7, respectively. These results indicate that IDF is easier to bind to nitrite under acidic conditions, and the combination of microwaves and enzymes can improve the adsorption capacity of IDF. As can be seen in Figure 6c, with increasing glucose concentration, the adsorption capacity of IDF obtained with different extraction methods gradually increased, and MEE-IDF had a higher glucose adsorption capacity than EE-IDF. As shown in Figure 6d, MEE-IDF had a stronger inhibitory ability on α-amylase than EE-IDF (62.46% vs. 43.92%, respectively). The reason may be that the multi-void structure of MEE-IDF is conducive to the adsorption of α-amylase on the surface of cellulose, so cellulose can act as an α-amylase inhibitor [47] and alleviate postprandial hyperglycemia [48]. The MEE-IDF showed increases in functional activity to some extent, which might be due to the generation of more pores and larger specific surface area under the joint action of microwaves and enzymes. Different extraction methods lead to different properties of IDF. Therefore, the optimal extraction method can be selected according to the actual demand in production and processing.

## 4. Conclusions

This study demonstrated that different extraction methods have significant effects on the physicochemical properties and functional activities of SDF and IDF extracted from PUP. MEE has a higher SDF yield, and MEE-SDF exhibits the highest adsorption capacity for cholesterol and nitrite in gastric juice under acidic conditions. TPP-SDF has the lowest molecular weight, loose and porous surface structure, the highest water holding capacity and thermal stability, and the strongest inhibition effect on glucose adsorption and α-amylase. Compared with EE-IDF, MEE-IDF has a better physicochemical property, thermal stability, and hypoglycemic activity in vitro. Taken together, MEE and TPP can promote the functional and structural properties of SDF and IDF extracted from PUP.

## Figures and Tables

**Table 1:** Dietary fiber components by different extraction methods.

Proximate Composition (g/100 g)	PUPP	EE-SDF	MEE-SDF	TPP-SDF	EE-IDF	MEE-IDF
Dietary fiber	79.90 ± 1.02 ^e^	96.23 ± 0.86 ^c^	97.45 ± 0.72 ^b^	96.63 ± 0.81 ^c^	95.78 ± 1.05 ^d^	97.96 ± 0.83 ^a^
Ash	0.80 ± 0.03 ^c^	0.67 ± 0.16 ^e^	0.75 ± 0.20 ^d^	0.55 ± 0.27 ^f^	1.18 ± 0.22 ^a^	1.06 ± 0.17 ^b^
Protein	2.21 ± 0.05 ^a^	1.14 ± 0.14 ^c^	1.09 ± 0.09 ^d^	0.85 ± 0.06 ^e^	1.28 ± 0.17 ^b^	1.26 ± 0.12 ^b^
Moisture	3.18 ± 0.07 ^a^	1.06 ± 0.12 ^e^	1.21 ± 0.10 ^d^	1.55 ± 0.11 ^b^	1.18 ± 0.07 ^b^	1.28 ± 0.14 ^c^
Total sugar	13.80 ± 0.17 ^d^	28.58 ± 0.14 ^c^	35.68 ± 0.22 ^b^	39.20 ± 0.17 ^a^	5.80 ± 0.16 ^f^	6.97 ± 0.20 ^e^
Yield (%)	-	5.80 ± 0.16 ^d^	7.68 ± 0.22 ^c^	3.97 ± 0.21 ^e^	71.21 ± 0.55 ^a^	67.81 ± 0.66 ^b^
WHC (g/g)	-	3.85 ± 0.23 ^d^	4.49 ± 0.54 ^b^	5.11 ± 0.29 ^a^	4.35 ± 0.15 ^c^	4.29 ± 0.18 ^c^
WBC (g/g)	-	3.40 ± 0.04 ^c^	3.54 ± 0.15 ^b^	4.05 ± 0.31 ^a^	0.94 ± 0.19 ^d^	0.88 ± 0.50 ^d^
WSC (mL/g)	-	2.03 ± 0.11 ^d^	2.93 ± 0.30 ^b^	3.48 ± 0.14 ^a^	1.67 ± 0.40 ^e^	2.20 ± 0.25 ^c^
OHC (g/g)	-	3.56 ± 0.22 ^d^	4.19 ± 0.10 ^b^	3.85 ± 0.33 ^c^	3.59 ± 0.13 ^d^	4.59 ± 0.29 ^a^

Note: PUPP, *P. ussuriensis* pomace powder; EE-SDF, soluble dietary fiber obtained by enzymic extraction; MEE-SDF, soluble dietary fiber obtained by microwave-assisted enzymatic extraction; TPP-SDF, soluble dietary fiber obtained by three-phase partitioning; EE-IDF, insoluble dietary fiber obtained by enzymic extraction; MEE-IDF, insoluble dietary fiber obtained by microwave-assisted enzymatic extraction. Results are expressed as means ± standard deviation (*n* = 3). Values with different letters in the same row are significantly different (*p* < 0.05).

**Table 2:** Monosaccharide composition of soluble dietary fiber by different extraction methods.

Monosaccharide Composition%	EE-SDF	MEE-SDF	TPP-SDF
Fuc	0.91 ± 0.13 ^b^	0.97 ± 0.14 ^b^	1.55 ± 0.21 ^a^
Rha	3.06 ± 0.14 ^c^	3.77 ± 0.17 ^b^	4.69 ± 0.25 ^a^
Ara	11.22 ± 0.47 ^c^	13.97 ± 0.56 ^b^	21.51 ± 0.82 ^a^
Gal	14.51 ± 0.64 ^b^	12.79 ± 0.59 ^c^	16.16 ± 0.71 ^a^
Glc	33.11 ± 2.13 ^a^	21.11 ± 1.66 ^b^	16.99 ± 1.07 ^c^
Xyl	4.54 ± 0.35 ^b^	3.76 ± 0.28 ^c^	6.20 ± 0.51 ^a^
Man	12.24 ± 0.65 ^b^	10.03 ± 0.59 ^c^	14.64 ± 0.67 ^a^
Fru	0.40 ± 0.05 ^a^	0.40 ± 0.02 ^a^	0.35 ± 0.03 ^b^
Rib	0.13 ± 0.04 ^c^	0.39 ± 0.06 ^a^	0.21 ± 0.04 ^b^
Gal-UA	19.41 ± 1.43 ^b^	32.39 ± 2.07 ^a^	16.87 ± 1.87 ^c^
Glc-UA	0.48 ± 0.11 ^b^	0.43 ± 0.08 ^b^	0.63 ± 0.07 ^a^
Man-UA	ND	ND	0.20 ± 0.01 ^a^

Note: EE-SDF, soluble dietary fiber obtained by enzymic extraction; MEE-SDF, soluble dietary fiber obtained by microwave-assisted enzymatic extraction; TPP-SDF, soluble dietary fiber obtained by three-phase partitioning. Results are expressed as means ± standard deviation (*n* = 3). Values with different letters in the same row are significantly different (*p* < 0.05).

**Table 3:** Molecular weights and polydispersity (Mw, Mn, and Mw/Mn) of SDFs.

Sample	Peak Number	RT (min)	Mn (Da)	Mw (Da)	D (Mw/Mn)	Area (%)
EE-SDF	1	11.74	2888	486,841	168.57	87.91
	2	12.77	31	135	4.35	10.17
MEE-SDF	3	8.22	12,081,875	20,760,280	1.72	4.27
	4	12.15	584	5432	9.28	92.90
TPP-SDF	5	11.32	5932	15,760	2.66	86.10
	6	12.79	119	185	1.55	5.48

Note: EE-SDF, soluble dietary fiber obtained by enzymic extraction; MEE-SDF, soluble dietary fiber obtained by microwave-assisted enzymatic extraction; TPP-SDF, soluble dietary fiber obtained by three-phase partitioning.

## Data Availability

Data presented in this article are available at request from the corresponding author.
